# Dr. William W. Smith

**Published:** 1917-03

**Authors:** 


**Dr. William W. Smith,** Bellevue 1871, died at his home in Avoca Feb. 4, aged 71.

